# Two new species of *Pachylaelaps* Berlese, 1888 from the Iberian Peninsula, with a key to European species (Acari, Gamasida, Pachylaelapidae)

**DOI:** 10.3897/zookeys.603.9038

**Published:** 2016-07-06

**Authors:** Peter Mašán, Hasan Hüseyin Özbek, Peter Fenďa

**Affiliations:** 1 Institute of Zoology, Slovak Academy of Sciences, Dúbravská cesta 9, 845-06 Bratislava, Slovakia; 2 Faculty of Science and Arts, Erzincan University, 24030 Erzincan, Turkey; 3 Department of Zoology, Faculty of Natural Sciences, Comenius University, Ilkovičova 6, 842-15 Bratislava, Slovakia

**Keywords:** Description, Europe, identification key, Mesostigmata, morphology, soil mites, systematics

## Abstract

Pachylaelaps (Pachylaelaps) pyrenaicus **sp. n.** and Pachylaelaps (Longipachylaelaps) brevipilis **sp. n.** (Acari, Pachylaelapidae) are described and illustrated based on specimens from litter and soil detritus of forest habitats in Spain (Pyrenees Mts) and Portugal (Serra da Labruja Mts), respectively. An identification key to European species of the genus *Pachylaelaps* Berlese, 1888 is provided.

## Introduction

Pachylaelapid mites (Acari, Mesostigmata, Gamasida, Eviphidoidea) represent a cosmopolitan group of free-living mites with extraordinarily wide ecological and behavioural diversity (including more than 230 known species and 16 genera worldwide). They constitute an important component of the fauna in all soil microhabitats of the temperate zone of the northern hemisphere. They colonise various soil substrates, especially leaf litter and decomposing organic detritus (Mašán and Halliday 2014).

The genus *Pachylaelaps* Berlese, 1888 belongs to the largest pachylaelapid genera and currently includes 56 valid species. Its continental diversity, based on the original type specimens and excluding those which have been incorrectly classified in the genus at some time, covers Europe (38 spp.), Asia (13 spp.), Africa (3 spp.), South America (1 sp.) and Australia (1 sp.) (Mašán and Halliday 2014, Özbek 2015). The genus was erected by Berlese (1888), placed in the Gamasidae by Berlese (1892) and *Gamasus pectinifer* G. & R. Canestrini, 1881 is generally accepted as its type species by subsequent designation by Berlese (1904) (see discussion of the type species by Mašán and Halliday (2014)). Later, a more comprehensive generic description was provided by Berlese (1904), Evans and Hyatt (1956), Costa (1971), and Koroleva (1977a) who classified the genus in the family Pachylaelapidae. Mašán (2007) clarified the concept of the genus *Pachylaelaps* by removing some species that obviously belong in other genera (e.g. *Onchodellus* Berlese, 1904 and *Pachydellus* Mašán, 2007), and described the new subgenus *Longipachylaelaps*.

When compared with other taxa of edaphic mesostigmatic mites, *Pachylaelaps* are relatively little-known in Europe. The almost identical appearance of individual species, which causes difficulties in species identification, may also explain the small number of papers exclusively devoted to the European species of the genus *Pachylaelaps* (Evans and Hyatt 1956, Hirschmann and Krauss 1965, Koroleva 1977b, 1978, Moraza and Peña 2005). The most recent review and general summary of *Pachylaelaps* species was by Mašán and Halliday (2014), with a checklist of world species.

The main aim of this paper is to describe two new soil-inhabiting species of the little known genus *Pachylaelaps*, compare them with other morphologically similar congeneric species, and provide an updated identification key to the European species of this genus.

## Materials and methods

Collected mites were extracted from the litter and soil detritus by means of a modified Berlese-Tullgren funnel equipped with a 40 Watt bulb, and preserved in ethyl alcohol. Before identification, the mites were mounted onto permanent microscope slides, using Swan’s chloral hydrate mounting medium. Illustrations were made by H. H. Özbek using a normal optical microscope equipped with a drawing tube. A Leica DM 1000 light microscope equipped with a stage-calibrated ocular micrometer and a Leica EC3 digital camera was used by P. Mašán to obtain measurements and photos. Measurements were made from slide-mounted specimens. Some multiple images were combined using the CombineZP software program (Hadley 2010). Lengths of shields and legs were measured along their midlines, and widths at their widest point (if not otherwise specified in the description). Dorsal setae were measured from the bases of their insertions to their tips. Measurements are mostly presented as ranges (minimum to maximum). The terminology of dorsal and ventral chaetotaxy follows Lindquist and Evans (1965). The notation for the pore-like structures of the idiosoma is that of Johnston and Moraza (1991).

## Systematics

### Genus Pachylaelaps

Taxon classificationAnimaliaMesostigmataPachylaelapidae

Berlese

Pachylaelaps Berlese, 1888: 196. Type species Gamasus pectinifer G. Canestrini, 1881, by subsequent designation (Berlese 1904).

#### Diagnosis.

*Pachylaelaps* can be reliably diagnosed by the following combination of characters: (1) dorsal shield oblong, suboval, and bearing 30 pairs of mostly subequal setae; (2) dorsocentral setae J2 in normal posterolateral position to setae J1; (3) sternal and genitoventral shield with four and two pairs of setae, respectively; (4) female tarsus II with two spur-like distal setae, pl1 and pl2; (5) sperm induction system of female associated with coxae IV; (6) tibial projections on male palp developed (except for species of *Pachylaelaps pectinifer* group); (7) genu I with 13 setae.

#### Taxonomic notes.

Mašán (2007) divided the genus into two subgenera, *Pachylaelaps* s. str. and *Longipachylaelaps* Mašán, 2007. That taxonomic concept is used also in this paper. The subgenus *Longipachylaelaps* is reliably distinguished from the subgenus *Pachylaelaps* s. str. mainly by the presence of normal needle-like dorsal setae J5, and only one pair of slit-like poroid structures (gdS4) placed on the posterolateral margin of the dorsal shield. In the subgenus *Pachylaelaps* s. str., setae J5 are vestigial, and the posterolateral dorsal shield margin bears two pairs of slit-like poroids, gdZ1 and gdS4.

### Pachylaelaps (Pachylaelaps) pyrenaicus sp. n.

Taxon classificationAnimaliaMesostigmataPachylaelapidae

http://zoobank.org/4E352060-75DC-4D92-A1FE-8BCDC82CD6B7

Figures 1–2 , 3–7 , 8–12 , 13–19

#### Specimens examined.

Holotype female: North Spain, Central Pyrenees Mts., Cinca Valley, Bielsa Cadaster, Salinas Village (near-by San Marcial Settlement), pine forest (*Pinus* spp.) with admixed beech (*Fagus sylvatica*), soil detritus with deep layer of raw humus between rock boulders, altitude 1050 m, 42°35'52,2"N, 00°14'20,0"E, 16 June 2007, coll. P. Fenďa. Paratypes: four females and one male, with the same data as the holotype. The holotype and four paratypes are deposited at the Institute of Zoology, Slovak Academy of Sciences, Bratislava; one female paratype is deposited at the Acarology Laboratory of Erzincan University, Turkey.

#### Diagnosis.

Slit-like glandular poroids gdZ1 and gdS4 with conspicuously adjacent position. Soft integument with decreased number of 11 pairs of setae in female and eight setal pairs in male. Prestigmatic section of peritreme long, with anterior tip reaching dorsal surface close to setae z1. Dorsal setae long (the longest setae more than 100 μm in length), and seta j5 with tip reaching base of following seta z5. Cheliceral digits unidentate. Male palptibia with two well developed petal-like projections. In female, ventrodistal femur with small spine-like process associated with a seta. Terminal part of male tarsus II with only one spur-like distal seta (pl1). Sperm induction system with tubular components: tubes irregularly formed, folded, curved or with small bumps on distal sections, progressively widened basally; basal part widely abutting to anterior margin of coxa IV.

#### Description.

**Figures 1–2.**
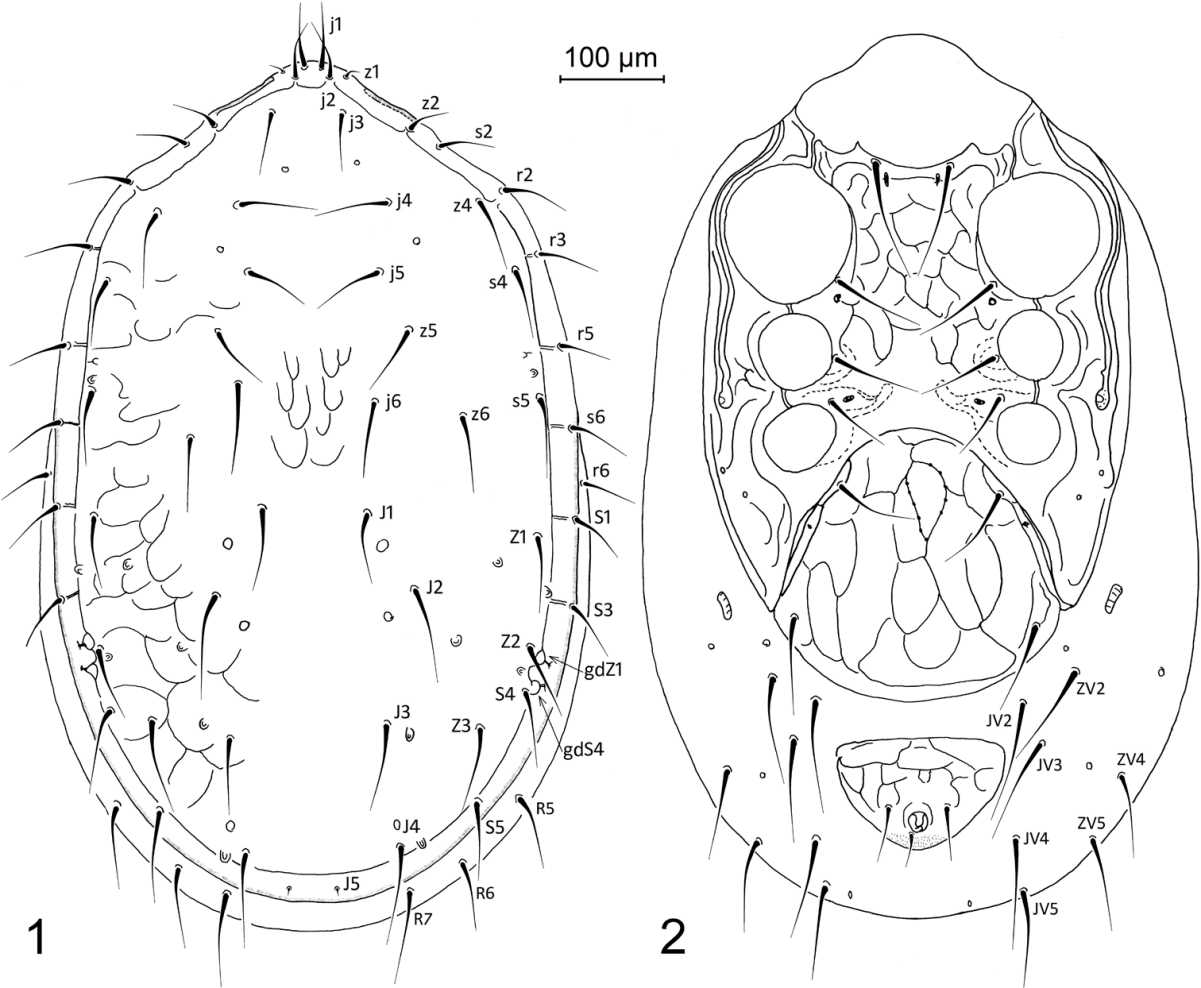
Pachylaelaps (Pachylaelaps) pyrenaicus, female, with setal notation of some idiosomal setae and glandular poroids. **1** Dorsal idiosoma **2** Ventral idiosoma.

*Female*. *Dorsal idiosoma* (Figure 1). Dorsal shield 870–915 μm long and 560–610 μm wide, suboval (length/width 1.48–1.63), weakly and unevenly reticulated on surface, and bearing 30 pairs of smooth and needle-shaped dorsal setae. Setae z1 conspicuously shortened, setae J5 strongly reduced in length, vestigial microsetae; other setae relatively longer, subequal and uniform. Length and spacing of some selected dorsal shield setae as follows: j1 53–67 μm, j5 73–83 μm, j5–j5 128–144 μm, j5–z5 72–81 μm, J1–J2 87–111 μm, J2 97–109 μm, J2–J2 216–242 μm, J2–J3 142–172 μm, J3 102–110 μm, J3–J4 118–151 μm, and J4 100–105 μm. Dorsolateral soft integument with four pairs of marginal setae (r6, R5‒R7). Posterolateral poroid structures gdZ1 and gdS4 slit-like, markedly adjacent each other, and placed close to setae Z2 or rarely between setae Z2 and S4.

*Ventral idiosoma* (Figure 2). Sternal shield 272–280 μm long, proportionally 0.82–0.94 shorter than genitiventral shield, with concave anterior margin and two small projections close to bases of sternal setae st1. Genitiventral shield slightly shorter than wide or subequal in length and width (length 295–335 μm, width 308–337 μm, length/width 0.94–0.99). Anal shield subtriangular, 114–137 μm long and 170–199 μm wide (length/width 0.62–0.73); anus with circum-anal setae situated close to posterior margin of shield. Peritremes well developed, relatively long, with anterior tip reaching dorsal surface close to setae z1. Peritrematal shields with weak longitudinal sculptural lines, other ventral shields distinctly and evenly reticulated on surface. Metapodal platelets minuscule, free and well separate from peritrematal shields. Ventral soft integument with seven pairs of ventral setae (JV2–JV5, ZV2, ZV4, ZV5). Ventral setae similar to those on dorsal idiosoma.

**Figures 3–7.**
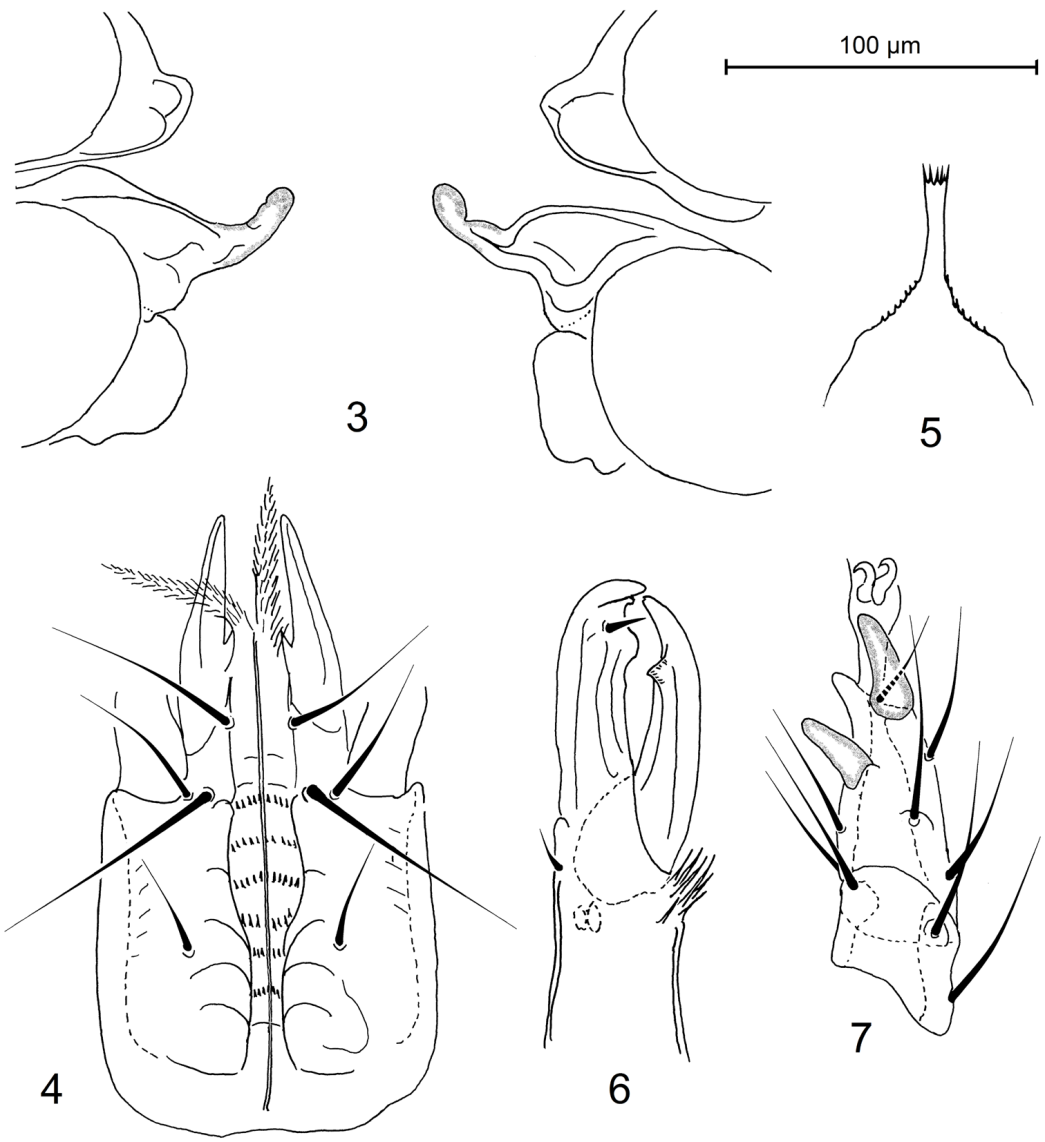
Pachylaelaps (Pachylaelaps) pyrenaicus, female. **3** Sperm induction structures **4** Ventral gnathosoma **5** Epistome **6** Chelicera, lateral view **7** Tarsus II, lateral view.

*Sperm induction structures* (Figures 3, 13‒16). Tubes of sperm induction system relatively well developed, well sclerotized, broadened basally, and narrowed distally; worm-like distal section irregularly formed, folded, curved or with small bumps; basal section widely abutting to anterior margin of coxa IV.

*Gnathosomal structures* (Figures 4‒6, 19). Corniculi elongated and horn-like; laciniae densely pilose, slightly longer than corniculi; deutosternum slightly widened medially, with six rows of denticles; subcapitular setae smooth and needle-shaped (Figure 4). Epistome with wide subtriangular base, elongate and narrow central neck and thin apical part crenelated on anterior margin; lateral margins of basal part with delicate denticulation; apical section not expanded or only very slightly expanded anteriorly, terminally truncate and with a row of four to seven prongs (Figure 5). Cheliceral digits relatively elongate and slender (Figures 6, 19), 100–110 μm long; fixed digit of chelicera with terminal hook, small and obtuse subapical denticle, and one larger and flattened distal tooth associated with pilus dentilis; movable digit armed with relatively thin terminal hook and one subdistal tooth.

*Legs*. Leg setation normal for genus (Mašán 2007). Femur II with a small spine-like process on ventral distal surface, process associated with a seta. Tarsus II with two spur-like distal setae pl1 and pl2 (Figure 7).

**Figures 8–12.**
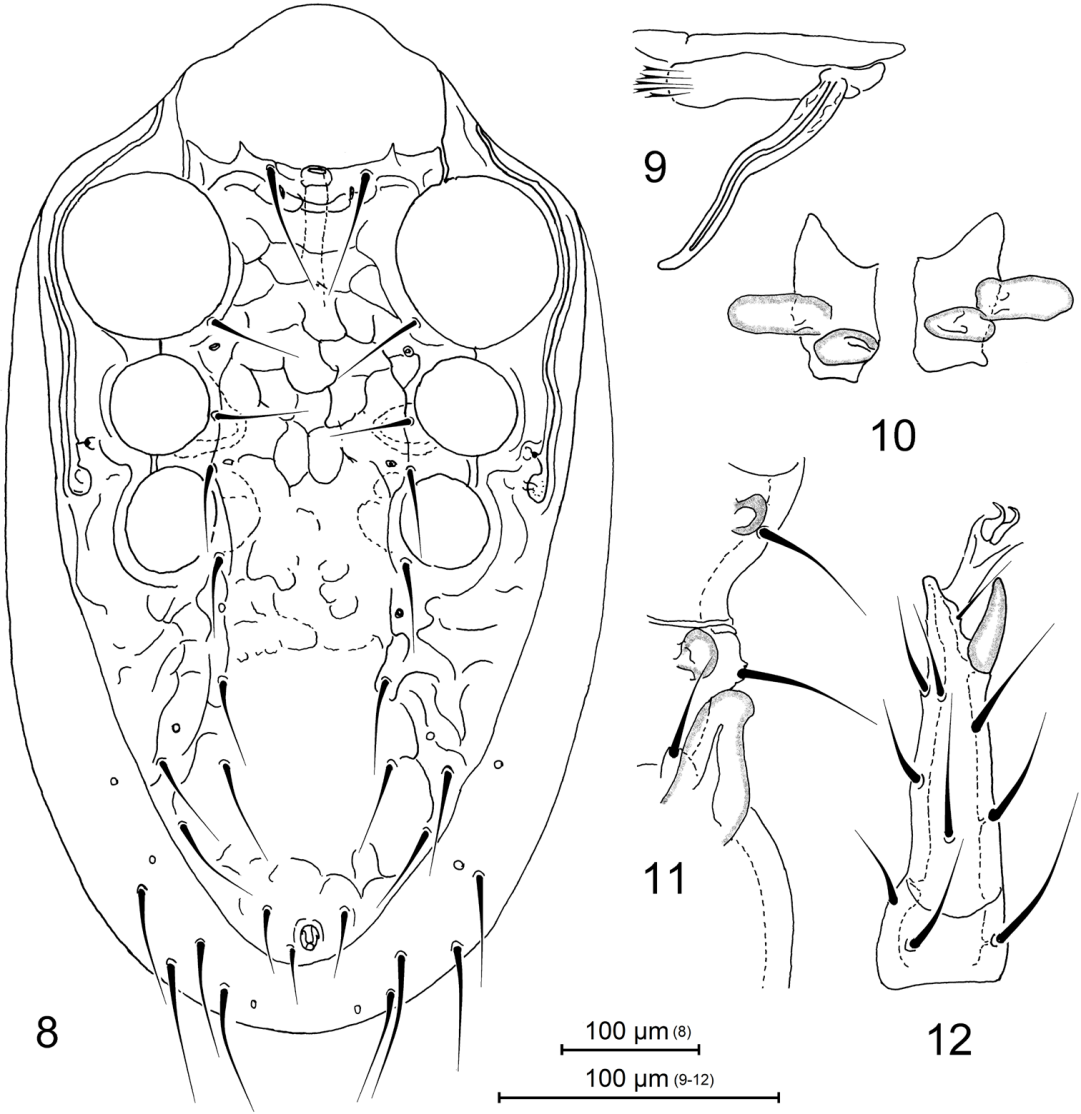
Pachylaelaps (Pachylaelaps) pyrenaicus, male. **8** Ventral idiosoma **9** Chelicera, ventrolateral view **10** Palptibial projections **11** Projections on medial segments of leg II, ventral view **12** Tarsus II, lateral view.

*Male*. *Idiosoma* (Figure 8). Dorsal shield 810 μm long and 492 μm wide, suboval (length/width 1.65). Sternal, genitiventral, peritrematal, metapodal, and anal plates are fused together to form an entire holoventral shield bearing nine pairs of setae (excluding three circum-anal setae); the shield irregularly reticulate on surface. Dorsolateral and ventral soft integument with eight pairs of setae (see diagnosis). Dorsal and ventral chaetotaxy and other characters as in female.

**Figures 13–19.**
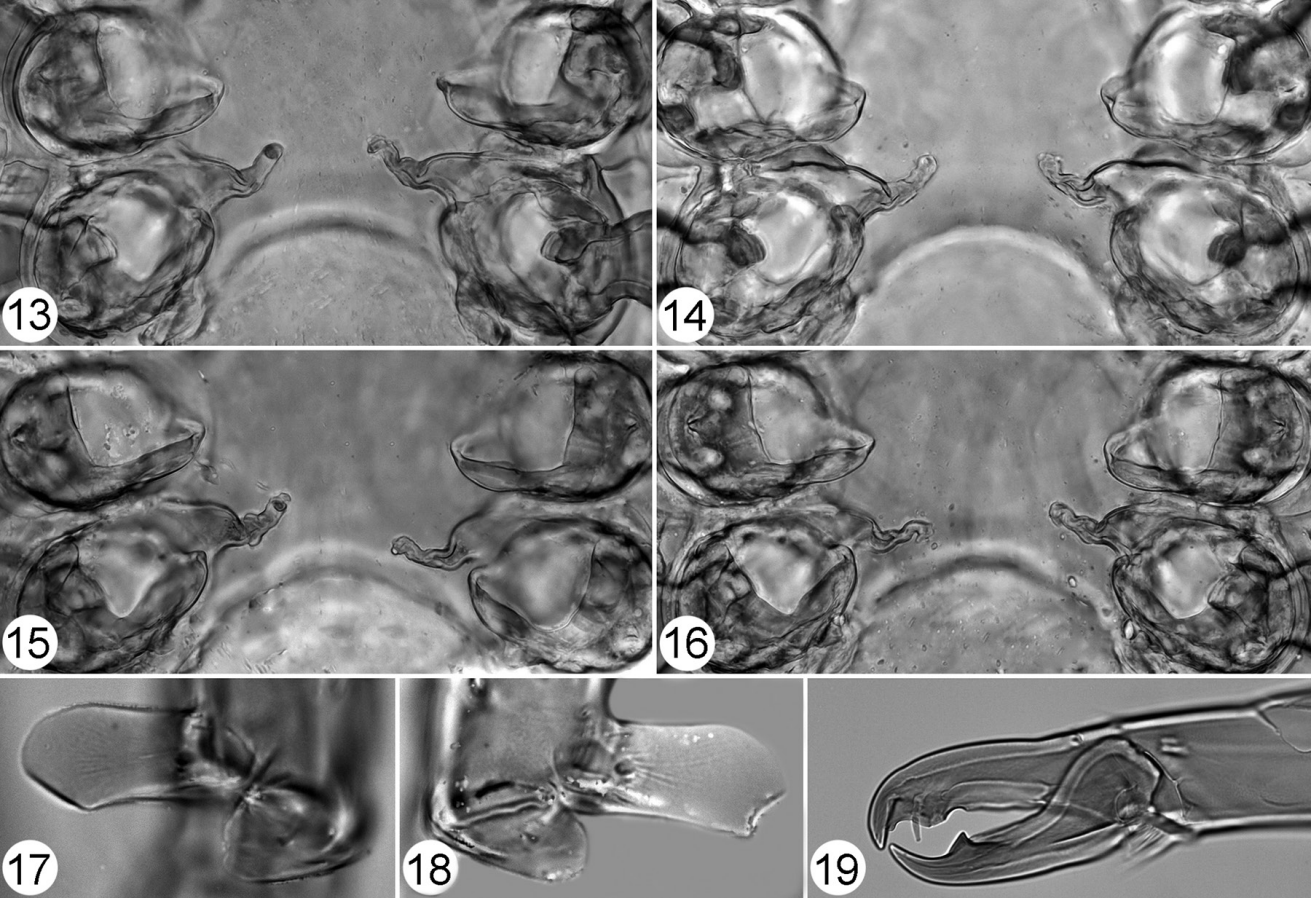
Pachylaelaps (Pachylaelaps) pyrenaicus. **13–16** Sperm induction structures, female, variant forms **17–18** Palptibial projections, male, variant forms **19** Chelicera, female, lateral view. Not scaled.

*Gnathosomal structures* (Figures 9, 10, 17, 18). Cheliceral spermatodactyl elongated, ensiform, 162 μm long (about 1.5 times as long as movable digit of chelicera), slightly widened in basal section, progressively tapering toward tip and slightly undulate medially; sperm ductus well defined (Figure 9). Palptibiae normal, not thickened (when compared with other palp segments), each bearing a pair of well-developed petal-like projections on proximal ventral surface (see Figures 17 and 18); outer petal markedly larger than inner one, and longer than cross-sectional radius of palptibia.

*Legs*. Medial segments of legs II spurred on their distal ventral surface: femur with one robust spur, genu and tibia each with a small knob-like spur, as in Figure 11. Femoral spur broadened basally, produced into obtuse and rounded apex; associated axillar seta pv1 inserted in a small tubercle (Figure 11). Terminal part of tarsus II with only one spur-like distal seta pl1; seta pl2 needle-shaped (Figure 12).

#### Etymology.

The epithet of this species is derived from the Latin name “*Pyrenaei Montes*” and alludes to the type locality situated in the Pyrenees Mountains.

#### Taxonomic notes.

The new species may be distinguished from all other congeners especially by the following combination of characters: (1) in female, tubiform spermathecal structures irregular, with worm-like distal sections having some bends, folds and small bumps, and expanded base widely abutting the anterior margin of coxa IV; (2) female chelicera with flat to truncate subdistal tooth on fixed digit; (3) epistome with narrow central projection bearing a row of four to six denticles; (4) male palptibia with two well developed petal-like projections; (5) terminal part of male tarsus II with only one spur-like distal seta, pl1 (6) cheliceral spermatodactyl simple, ensiform, slightly undulate medially, without irregular convexities or projections on its margin.

Mašán (2007) divided the European members of the subgenus *Pachylaelaps* into five clusters of species: (1) the *bellicosus* group (*Pachylaelaps bellicosus* and *Pachylaelaps multidentatus*), with separate position of slit-like poroid structures gdZ1 and gdS4 on dorsal shield, multidentate cheliceral digits, spermathecal tubiform structures simple, transparent (weakly sclerotized) and relatively longer, and males apparently absent; (2) the *denticulatus* group (*Pachylaelaps denticulatus* only), possessing separate position of slit-like poroid structures gdZ1 and gdS4, three projections on male palptibia, one spur-like distal seta on tarsus II in male, and bidentate cheliceral digits; (3) the *ensifer* group (*Pachylaelaps armimagnus*, *Pachylaelaps carpathimagnus*, *Pachylaelaps ensifer*, *Pachylaelaps troglophilus* and *Pachylaelaps sacculimagnus*), characterized by the adjacent position of slit-like poroid structures gdZ1 and gdS4, robust size of idiosoma, spermathecal tubiform structures (if detectable) elongated and weakly sclerotized, and presence of 2‒4 palptibial projections in male and two spur-like distal setae on tarsus II in both adults; (4) the *imitans* group (*Pachylaelaps imitans*, *Pachylaelaps insularis*, *Pachylaelaps resinae* and *Pachylaelaps terreus*), with adjacent slit-like poroid structures gdZ1 and gdS4 having their openings in a common infundibulum, spermathecal tubiform structures short, conical to cylindrical and strongly sclerotized, two palptibial projections in male, and small lobe-like convexity on ventral margin of cheliceral spermatodactyl; (5) the *pectinifer* group (*Pachylaelaps littoralis* and *Pachylaelaps pectinifer*), characterized by the adjacent position of slit-like poroid structures gdZ1 and gdS4, Y-shaped spermathecal tubiform structures, absence of palptibial projections in male, and presence of two spur-like distal setae on tarsus II in both adults.

In this classification scheme, Pachylaelaps (Pachylaelaps) pyrenaicus should be considered as a species with a separate position among the all above mentioned species groups because it possesses a unique combination of main diagnostic characters. Some morphological characters of Pachylaelaps (Pachylaelaps) pyrenaicus are not consistent with those found typically in the individual species groups. The adjacent position of slit-like poroid structures gdZ1 and gdS4 on dorsal shield and unidentate cheliceral digits in the new species are in contradiction with the definition of the *bellicosus* and *denticulatus* groups. The male palptibia has two petal-like projections in Pachylaelaps (Pachylaelaps) pyrenaicus, where the *pectinifer* group species does not have these structures developed. In the robust species of the *ensifer* group, tarsus II has two spur-like distal setae in adults, but this character is found in the smaller new species only in females. In addition, in Pachylaelaps (Pachylaelaps) pyrenaicus, the tubular structures of sperm induction system have a distinctive form which is not known in the other species of the genus, but is especially different from the members of the *imitans* group that are characterized by short, conical to cylindrical, and strongly sclerotized spermathecal tubes.

### Pachylaelaps (Longipachylaelaps) brevipilis sp. n.

Taxon classificationAnimaliaMesostigmataPachylaelapidae

http://zoobank.org/89C70A15-424B-4D59-9D17-E4642314663B

Figures 20–21 , 22–26 , 27–34 , 35–38 , 39–42

#### Specimens examined.

Holotype female: North Portugal, Serra da Labruja Mts., San Bento da Porta Aberta Village, Viana do Castelo Cadaster, non-native eucalyptus forest (*Eucalyptus globulus*), humid leaf litter and soil detritus, altitude 260 m, 41°56'02,3"N, 08°37'49,9"W, 10 May 2008, coll. P. Fenďa. Paratypes: 45 females and 14 males, with the same data as in holotype. The holotype and paratypes are deposited at the Institute of Zoology, Slovak Academy of Sciences, Bratislava; six paratypes (three females and three males) are deposited at the Acarology Laboratory of Erzincan University, Turkey.

#### Diagnosis.

Soft integument with decreased number of 13 pairs of setae in female and ten setal pairs in male. Dorsal setae J5 well developed, slightly longer than setae J4. Prestigmatic section of peritreme long, with anterior tip reaching dorsal surface close to setae z1. Dorsal setae relatively short (longest setae not exceeding 35 μm in length), with their tips not reaching bases of following setae. Cheliceral digits unidentate; pilus dentilis conspicuously enlarged (in female) or vestigial (in male). Male palptibia with two petal-like projections, shorter than cross-sectional radius of palptibia. Terminal part of male tarsus II with only one spur-like distal seta (pl1). Sperm induction system with tubular components: tubes relatively shorter, with club-shaped apical section, straight or variously curved; basal part not markedly expanded, thin, associated with inner middle surface of coxa IV.

#### Description.

**Figures 20–21.**
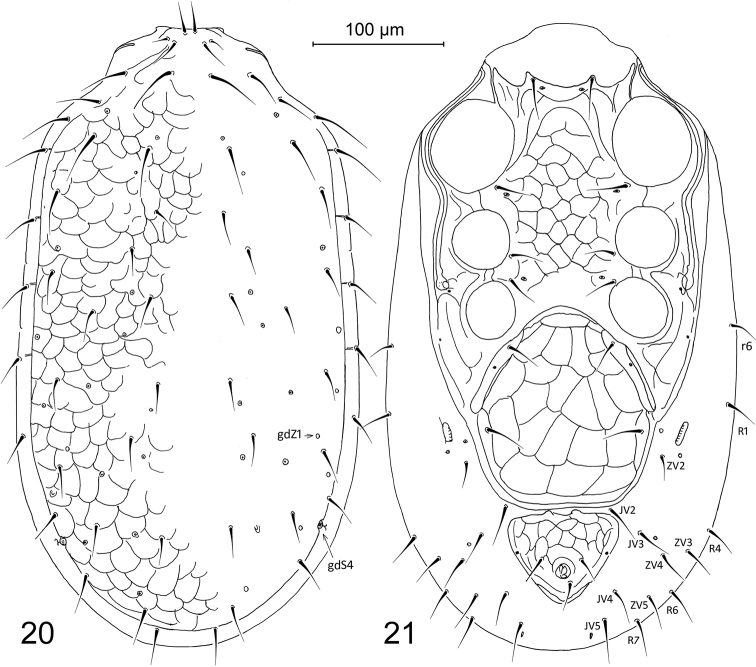
Pachylaelaps (Longipachylaelaps) brevipilis, female, with setal notation of some ventral setae and glandular poroids. **20** Dorsal idiosoma **21** Ventral idiosoma.

*Female*. *Dorsal idiosoma* (Figure 20). Dorsal shield 510–565 μm long and 285–315 μm wide, elongated and suboval (length/width 1.75–1.95), delicately and evenly reticulated on surface, and bearing 30 pairs of dorsal setae. Dorsal setae uniform, smooth and needle-shaped, subequal in length, relatively short, and mostly with tips not reaching bases of following setae; setae z1 shortest and setae j3, j4, z4, r2, and r3 longest (46–51 μm). Length and spacing of some selected dorsal shield setae as follows: j1 24–29 μm, j5 22–26 μm, j5–j5 53–64 μm, j5–z5 31–41 μm, J1 25–29 μm, J1–J2 44–54 μm, J2 26–31 μm, J2–J2 98–114 μm, J2–J3 88–97 μm, J3 24–30 μm, J3–J4 63–85 μm, J4 22–30 μm, and J5 24–31 μm; setae J4/J5 0.87–0.96. Dorsolateral soft integument with five pairs of marginal setae (r6, R1, R4, R6, R7). One pair of posterolateral poroid structures (gdS4) slit-like, placed between setae S4 and S5.

*Ventral idiosoma* (Figure 21). Sternal shield 190–205 μm long, usually longer than genitiventral shield (length of sternal shield/length of genitiventral shield 0.98–1.12), with concave anterior margin and two small corners close to bases of sternal setae st1. Genitiventral shield slightly longer than wide (length 175–202 μm, width 155–185 μm, length/width 1.05–1.17). Anal shield subtriangular, 70–85 μm long and 95–115 μm wide (length/width 0.65–0.80); anus with circum-anal setae situated close to posterior margin of shield. Peritremes well developed, with anterior tip reaching dorsal surface between setae z1 and z2. Peritrematal shields with weak longitudinal sculptural lines, other ventral shields distinctly and evenly reticulated on surface. Metapodal platelets minuscule, free on soft integument, and situated at level of setae JV1. Ventral soft integument with eight pairs of ventral setae (JV2–JV5, ZV2–ZV5). Ventral setae similar to those on dorsal idiosoma.

**Figures 22–26.**
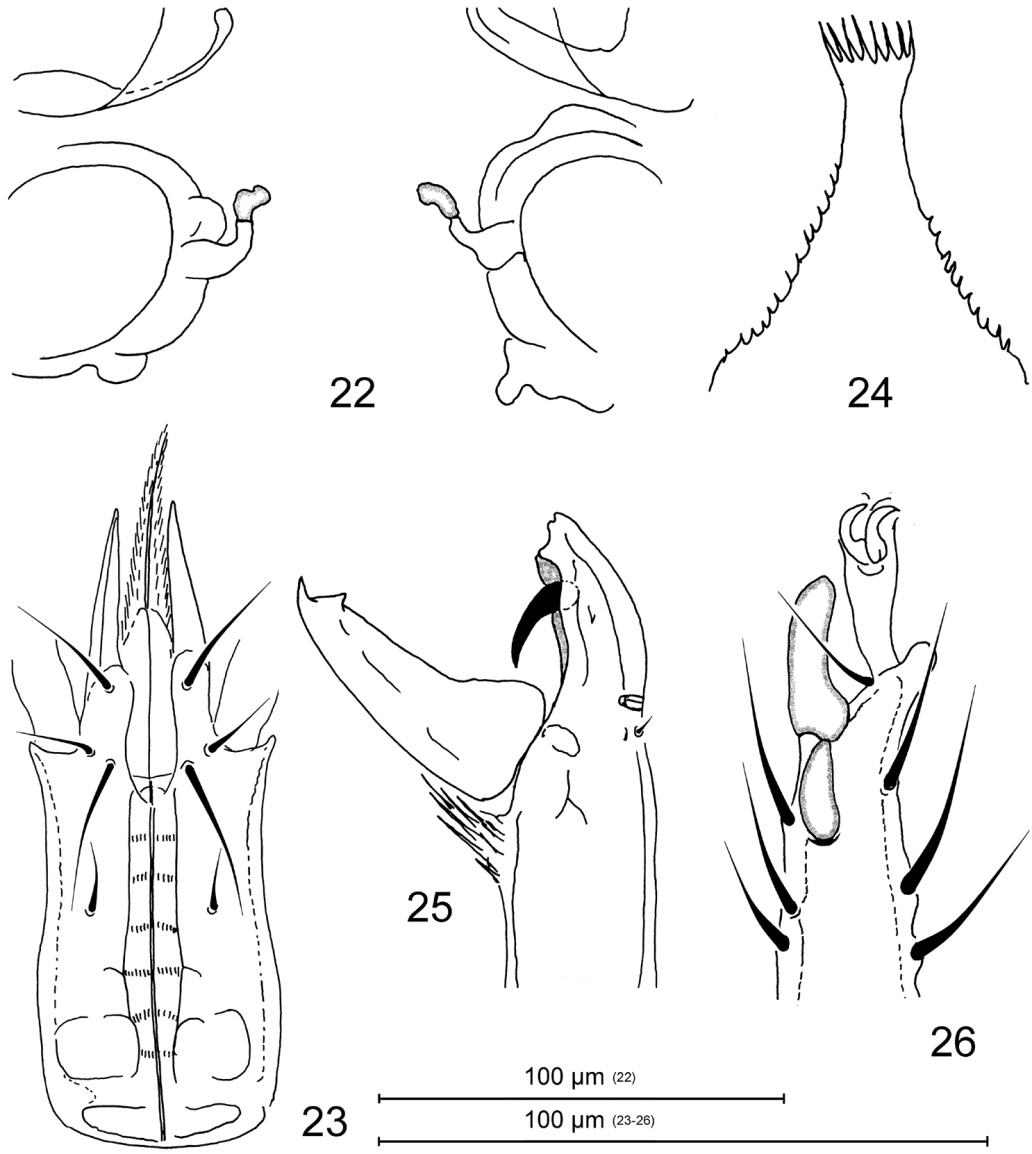
Pachylaelaps (Longipachylaelaps) brevipilis, female. **22** Sperm induction structures **23** Ventral gnathosoma **24** Epistome **25** Chelicera, lateral view **26** Tarsus II, posterolateral view.

**Figures 27–34.**
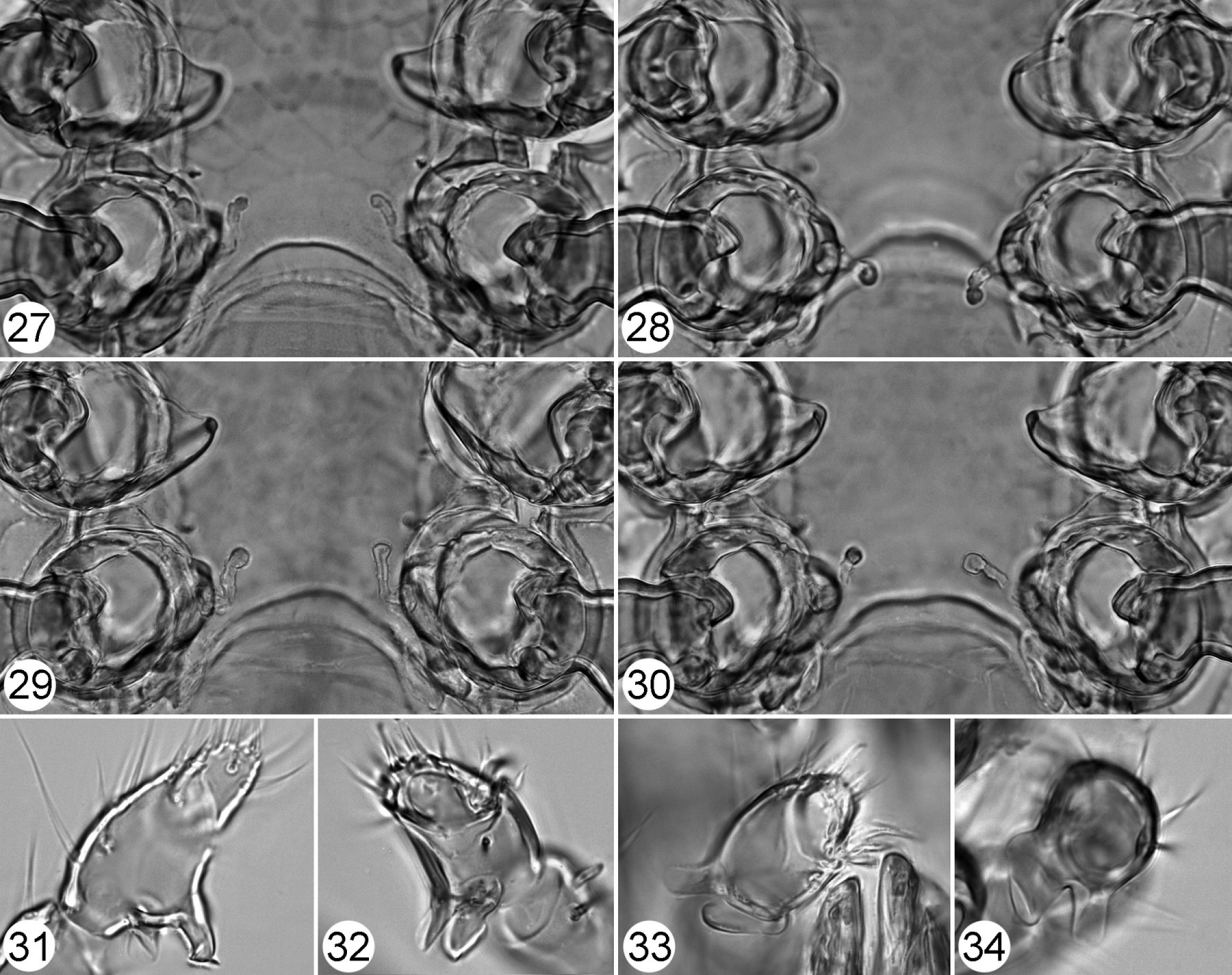
Pachylaelaps (Longipachylaelaps) brevipilis. **27–30** Sperm induction structures, female, variant forms **31–34** Palptibial projections, male, variant views. Not to scale.

*Sperm induction structures* (Figures 22, 27‒30). Tubes of sperm induction system weakly developed (with well-separated tips), weakly sclerotized in narrow basal and medial part, broadened apically, and club-shaped; basalmost section connected to inner margin of coxa IV; in newly moulted specimens, bulbiform apex of tubes more or less reduced, and almost hyaline (unsclerotised).

**Figures 35–38.**
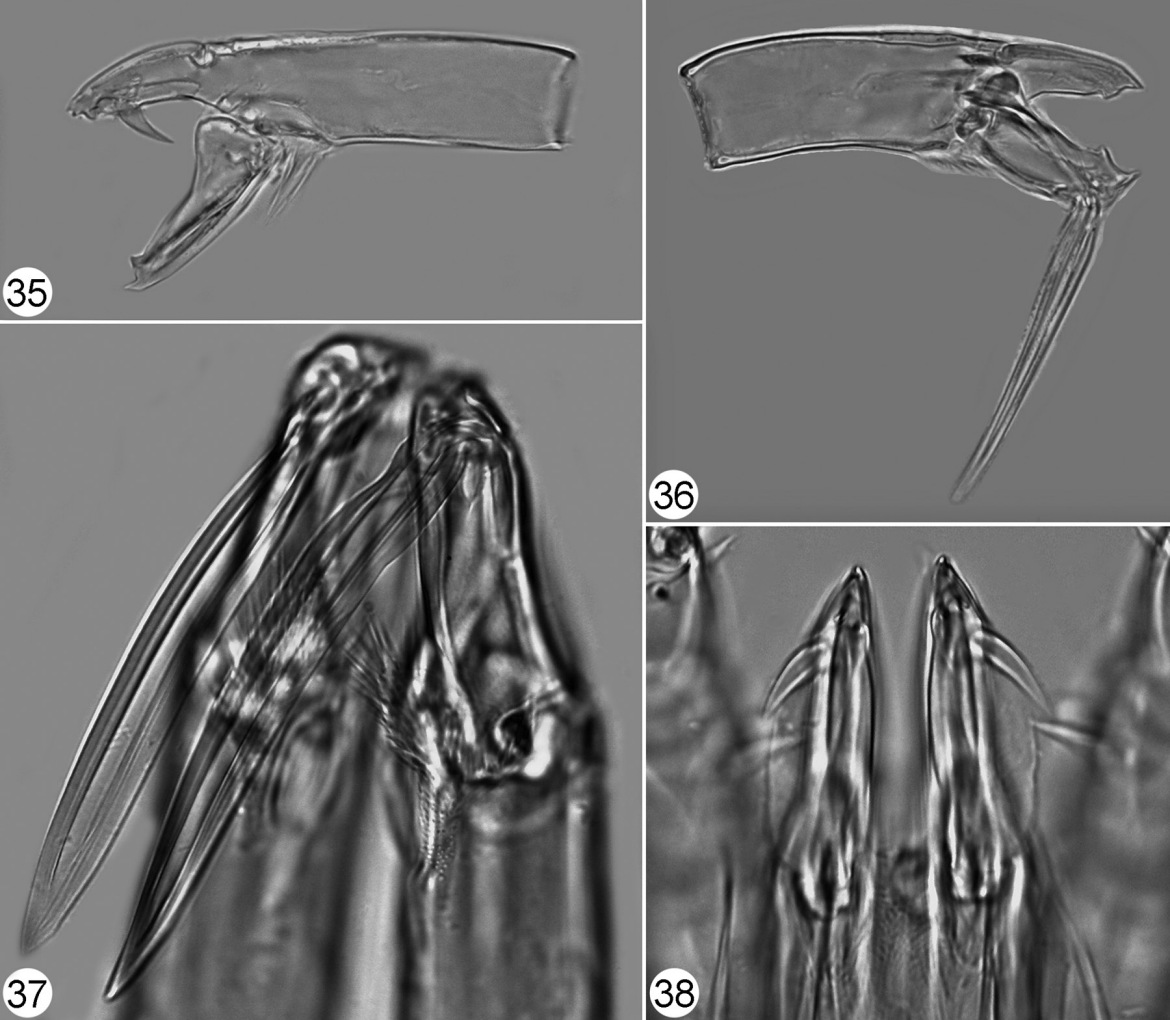
Pachylaelaps (Longipachylaelaps) brevipilis. **35** Chelicera, female, lateral view **36** Chelicera, male, lateral view **37** Chelicerae, male, ventrolateral view **38** Chelicerae, female, ventral view. Not to scale.

*Gnathosomal structures* (Figures 23‒25, 35, 38). Corniculi elongated and horn-like; laciniae densely pilose, longer than corniculi; deutosternum with six rows of denticles; subcapitular setae smooth and needle-shaped (Figure 23). Epistome with subtriangular and regularly narrowed base, wider central neck and widened apical part densely crenelated on truncate anterior margin; basal part serrate on lateral margins (Figure 24). Fixed digit of chelicera shortened, seemingly truncate; with indistinctive terminal hook reduced to two small denticles, one subdistal tooth, and very robust (hypertrophied) pilus dentilis directed backward (Figures 25, 35, 38). Movable digit of chelicera longer than fixed digit, with a hook and one subapical tooth (Figure 35).

*Legs.* Leg setation normal for genus (Mašán, 2007). Tarsus II with two spur-like distal setae, pl1 and pl2 (Figure 26).

**Figures 39–42.**
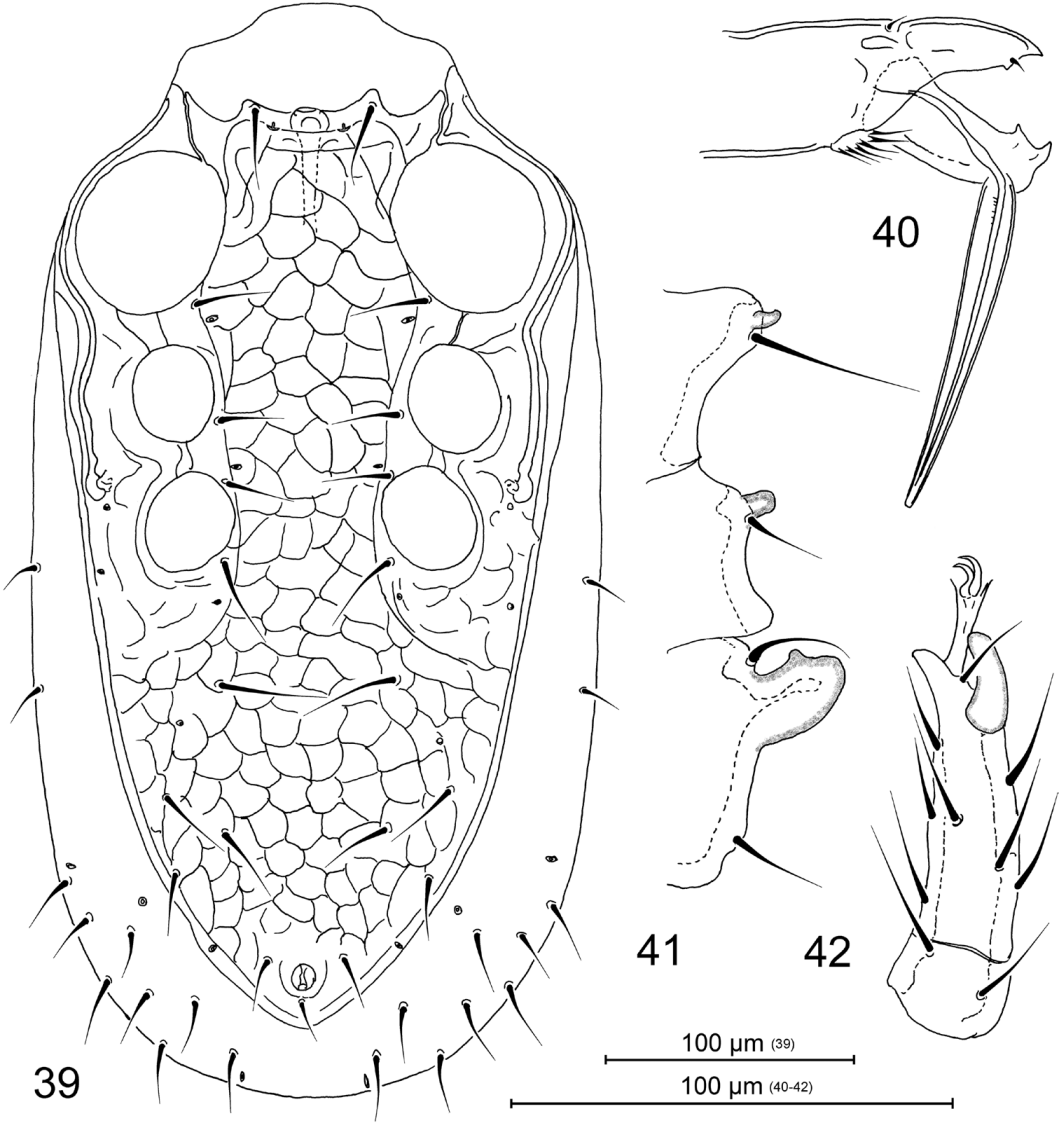
Pachylaelaps (Longipachylaelaps) brevipilis, male. **39** Ventral idiosoma **40** Chelicera, lateral view **41** Projections on medial segments of leg II, lateral view **42** Tarsus II, posterolateral view.

*Male*. *Idiosoma* (Figure 39). Dorsal shield 470‒510 μm long and 250‒285 μm wide, elongated and suboval (length/width 1.78‒1.88). Sternal, genitiventral, peritrematal, metapodal, and anal plates fused together to form an entire holoventral shield bearing nine pairs of setae (not including three circum-anal setae); shield evenly reticulate on surface. Dorsolateral and ventral soft integument with ten pairs of setae (see diagnosis). Dorsal and ventral chaetotaxy, and other characters as in female.

*Gnathosomal structures* (Figures 31‒34, 36, 37, 40). Palptibiae slightly thickened medially (when compared with other palp segments), each bearing a pair of petal-like projections on proximal ventral surface, as in Figures 31‒34; inner petal markedly larger than outer one, but shorter than cross-sectional radius of palptibia. Cheliceral spermatodactyl elongated, ensiform, 75‒80 μm long (about 1.7‒1.9 times as long as movable digit of chelicera), slightly widened in proximal section and progressively tapering toward the tip; sperm ductus well defined (Figures 36, 37, 40).

*Legs.* Medial segments of legs II spurred on their distal ventral surface: femur with one robust spur, genu and tibia each with a peg-like spur, as in Figure 41. Femoral spur broadened medially, produced into widely rounded apex, with a small subdistal tubercle (Figure 41). Terminal part of tarsus II with only one spur-like distal seta, pl1 (Figure 42).

#### Etymology.

The specific name of the new species is derived from the Latin words “*brevis*” (short) and “*pilum*” (hair), and it alludes to the fact that the species has the shortest idiosomal setae among its congeners.

#### Taxonomic notes.

The main diagnostic character states for Pachylaelaps (Longipachylaelaps) brevipilis are the presence of shorter idiosomal setae (e.g., j5 < j5–z5, J1 ≈ ½ x J1–J2), the relative length of dorsal setae J4 and J5 (setae J5 negligibly longer than J4, about 1.04–1.14 times as long as J4), the existence of sexual dimorphism in the pilus dentilis (markedly enlarged and spiniform in female, minute and slender in male), the form of the tubular structures of the sperm induction system (tubes shorter, with club-like terminal part), the form and length of the cheliceral spermatodactyl (sword-like, less than twice as long as movable digit), and the length of the dorsal shield (small species, with dorsal shield 470–510 μm long in males, and 510–565 μm long in females).

The presence of relatively short dorsal setae (at least in a central row), along with subequal setae J4 and J5, is also the feature of three other Pachylaelaps (Longipachylaelaps) species, namely Pachylaelaps (Longipachylaelaps) bifurciger, Pachylaelaps (Longipachylaelaps) dubius and Pachylaelaps (Longipachylaelaps) silviae. The new species may be reliably distinguished from the above mentioned congeners by the characters presented in Table 1, and with the help of the identification key provided below.

**Table 1.** Comparative characteristics of the females of four similar species of the subgenus Pachylaelaps (Longipachylaelaps).

Character / Species	Pachylaelaps (Longipachylaelaps) brevipilis sp. n.	Pachylaelaps (Longipachylaelaps) bifurciger	Pachylaelaps (Longipachylaelaps) dubius	Pachylaelaps (Longipachylaelaps) silviae
Length of dorsal shield	510‒565 μm	910 μm	unknown	836 μm
Length of seta J1	J1 ≈ ½ J1‒J2	J1 ≈ ½ x J1‒J2	J1 ≥ J1‒J2	J1 < J1‒J2
Length of seta z6	z6 < ½ z6‒Z1	z6 ≈ ½ x z6‒Z1	z6 ≤ z6‒Z1	z6 > z6‒Z1
Length of seta J3	J3 < ½ J3‒J4	J3 < ½ J3‒J4	J3 ≈ J3‒J4	J3 < J3‒J4
Pilus dentilis	enlarged, spiniform	unknown	enlarged, spiniform	minute, setiform
Genitoventral shield	longer than wide (1.05‒1.17)	wider than long (0.87)	longer than wide (1.18)	as long as wide (≈ 1)
Terminal epistome	densely crenelated	bifurcate	unknown	densely crenelated
Spermathecal tubes	club-like distally, shorter (tips distant)	worm-like distally, longer (tips adjacent)	unknown (not detectable ?)	strongly elongated, spirally convoluted

### Key to European species of the genus *Pachylaelaps* (females)

Partial keys to the European species of *Pachylaelaps* may be found in Hirschmann and Krauss (1965), Karg (1971, 1993), Koroleva (1977a), and Mašán (2007). The identification of *Pachylaelaps* species is complicated by the inaccurate and inadequate descriptions of some species. Mašán (2007) and Mašán and Halliday (2014) attempted to clarify the concept of the genus by removing many species that obviously belong in other genera such as *Onchodellus* Berlese, 1904 and *Pachydellus* Mašán, 2007.

Due to vague and inadequate original descriptions, the particular structures of the sperm induction system, palptibial outgrowths and some other important characters remain unknown in a large number of species. Therefore several species are not included in the keys presented in this paper, namely Pachylaelaps (Pachylaelaps) bicornis Willmann, 1939 (♀), Pachylaelaps (Longipachylaelaps) dubius Hirschmann & Krauss, 1965 (♀), Pachylaelaps (Pachylaelaps) grandis Koroleva, 1977 (♀), Pachylaelaps (Longipachylaelaps) granulifer Hirschmann & Krauss, 1965 (♀, but ♂ included), Pachylaelaps (Longipachylaelaps) longisetis Halbert, 1915 (♂, but ♀ included), Pachylaelaps (Longipachylaelaps) obirensis Schmölzer, 1992 (♀, ♂).

**Table:** 

1	Dorsal setae J5 developed, needle-like; posterolateral margins of dorsal shield with one pair of slit-like poroid structures, gdS4 (subgenus *Longipachylaelaps* Mašán, 2007)	**2**
–	Setae J5 vestigial; posterolateral margins of dorsal shield with two pairs of slit-like poroid structures, gdZ1 and gdS4 (subgenus *Pachylaelaps* s. str.)	**15**
2	Structures of sperm induction system between coxae IV well sclerotized (at least some basal or distal components), usually well discernible and striking in their lines	**3**
–	Structures of sperm induction system weakly sclerotized, hyaline and transparent, scarcely detectable, often poorly developed or fully reduced	**12**
3	Sperm induction system tubular: tubiform structures simple, short or elongated, straight or curved, sometimes variously convoluted, or broadened distally	**4**
–	Sperm induction system sacculate or otherwise modified: tubiform structures absent and transformed into saccules with short tubiform opening only slightly protuberant above enlarged base, or into specific sickle-shaped structures	**10**
4	Tubiform structures short and straight; movable digit of chelicera with three separate dents: distal hook (often with tiny lateral denticle), and subdistal and medial teeth; length of dorsal shield 750–800 μm	***Pachylaelaps longisetis* Halbert, 1915**
–	Tubiform structures longer or otherwise formed; movable digit of chelicera with two separate dents: simple or bifid distal hook (often with tiny lateral denticle), and a subdistal tooth	**5**
5	Tubiform structures of sperm induction system intermediate in length (with their apices sufficiently separated)	**6**
–	Tubiform structures long (with distalmost sections adjacent)	**8**
6	Dorsal setae short: setae J1 with tips reaching between insertions of setae J1 and J2; setae J4 slightly shorter than setae J5 (J4/J5 0.87–0.96); pilus dentilis conspicuously enlarged, spine-like; smaller species, length of dorsal shield 510–565 μm	***Pachylaelaps brevipilis* sp. n.**
–	Dorsal setae long: setae J1 with tips reaching beyond insertions of setae J2; setae J4 at least 1.5 longer than setae J5; pilus dentilis normal, slender; larger species, dorsal shield at least 680 μm in length	**7**
7	Tubiform structures regularly sclerotized; pilus dentilis minute, with upright position; dorsal setae J5 markedly shortened, about 5–6 times shorter than setae J4; genitiventral shield 260–320 μm wide; length of dorsal shield 770–890 μm	***Pachylaelaps sublongisetis* Koroleva, 1977**
–	Medial portion of tubiform structures unsclerotized, hyaline; pilus dentilis larger, curved and directed backward; setae J5 less shortened, about 1.5–2.5 times shorter than setae J4; genitiventral shield 188–225 μm wide; length of dorsal shield 680–805 μm	***Pachylaelaps longulus* Willmann, 1938**
8	Tubiform structures excessively elongated, slightly tapered distally and helically convoluted (with 2–3 bends); length of dorsal shield 836 μm	***Pachylaelaps silviae* Moraza & Peña, 2005**
–	Tubiform structures normal in length, straight or curved, and moderately broadened distally	**9**
9	Dorsal setae J5 shortened, about 4–7 times shorter than setae J4; length of dorsal shield 870 μm	***Pachylaelaps squamifer* Berlese, 1920**
–	Setae J5 and J4 subequal in length; length of dorsal shield unknown	***Pachylaelaps pulsator* Hirschmann & Krauss, 1965**
10	Sperm induction system with specific sickle-shaped structures; pilus dentilis relatively robust, spine-like and directed backward; movable cheliceral digit with simple distal hook; genitiventral shield relatively narrower (length/width 1.14–1.27); length of dorsal shield 745–840 μm	***Pachylaelaps distinctus* Mašán, 2007**
–	Sperm induction system sacculate: saccules with short tubiform opening slightly protuberant above enlarged base; pilus dentilis minute, with upright position; movable cheliceral digit with bifid distal hook; genitiventral shield relatively wider (length/width 0.92–1.12)	**11**
11	Sperm ductus inside saccules relatively shorter, straight and directed to anterior margin of coxa IV; base of saccules abutting the coxa IV; dorsal setae J5 30–39 μm long, about 2–3.5 times shorter than setae J4; genitiventral shield relatively narrower (length/width 1.03–1.12); length of dorsal shield 745–885 μm	***Pachylaelaps vicarius* Mašán, 2007**
–	Sperm ductus inside saccules relatively longer, slightly curved and directed between coxae III and IV; base of saccules slightly widened, abutting the coxae III and IV; setae J5 20–25 μm long, about 5–7 times shorter than setae J4; genitiventral shield relatively wider (length/width 0.9–0.95); length of dorsal shield 940–1,050 μm	***Pachylaelaps bocharovae* Koroleva, 1978**
12	Tubiform structures of sperm induction system elongated (with more or less adjacent tips), straight or slightly curved	**13**
–	Tubiform structures shortened (with well separated tips) or not detectable	**14**
13	Tubiform structures thin and long, worm-like; margins of genitiventral and anal shield straight and markedly separated; dorsal setae J4 and J5 short and subequal in length; epistome with distal projection narrow and bifurcate apically (often with small denticle between lateral cusps); length of dorsal shield 910 μm	***Pachylaelaps bifurciger* Berlese, 1920**
–	Tubiform structures broadened, with slightly club-like tip; margins of genitiventral and anal shield undulate and closely abutting each other; setae J5 30–33 μm long, about 4–4.5 times shorter than setae J4; distal projection of epistome wide and densely crenelated anteriorly; length of dorsal shield 780–840 μm	***Pachylaelaps undulatus* Evans & Hyatt, 1956**
14	Tubiform structures short, broad, conical and delicately striated transversally; pilus dentilis relatively well developed, directed backward; dorsal setae J5 47–61 μm long, about 1.5–2 times shorter than setae J4; length of dorsal shield 685–835 μm	***Pachylaelaps carpathicus* Mašán, 2007**
–	Tubiform structures not detectable (only rudimentary and tenuous structures rarely present); pilus dentilis small, with upright position; setae J5 15–25 μm long, about 3.5–6.5 times shorter than setae J4; length of dorsal shield 740–930 μm	***Pachylaelaps perlucidus* Mašán, 2007**
15	Two slit-like poroid structures well separated on posterolateral dorsal surface: gdZ1 situated between setae Z1–Z2 (close to Z2) and gdS4 between setae S4–S5 (close to S4)	**16**
–	Two slit-like poroid structures with more adjacent position on posterolateral dorsal surface: gdZ1 and gdS4 situated between setae Z2 and S4, or close to setae Z2	**18**
16	Cheliceral digits bidentate; length of dorsal shield 880 μm	***Pachylaelaps denticulatus* Hirschmann & Krauss, 1965 *sensu* Koroleva, 1977**
–	Cheliceral digits multidentate: movable digit with 7–12 denticles	**17**
17	Lateromarginal and ventral soft integument with 11 pairs of setae; tubiform structures tenuous, worm-like and hyaline (hardly discernible); length of dorsal shield 615–670 μm	***Pachylaelaps multidentatus* Evans & Hyatt, 1956**
–	Lateromarginal and ventral soft integument with 14 pairs of setae; tubiform structures relatively broad, tapered apically, directed posteromedially, weakly sclerotized but well discernible; length of dorsal shield 650–750 μm	***Pachylaelaps bellicosus* Berlese, 1920**
18	Tubiform structures of sperm induction system Y-shaped, with greatly widened bases, straight and tubular distal sections, and subglobular teat-like apices; basal part V-shaped, with well sclerotized sides	**19**
–	Tubiform structures otherwise formed or not detectable	**20**
19	Openings of slit-like poroids gdZ1 and gdS4 closely adjacent; sternal surface with transversal linear pattern; genitiventral shield longer than wide (length/width 1.05–1.2); length of dorsal shield 720–970 μm	***Pachylaelaps littoralis* Halbert, 1915**
–	Openings of slit-like poroids gdZ1 and gdS42 relatively separate; sternal region with transversal-longitudinal linear pattern; genitiventral shield usually subequal in length and width (length/width 0.95–1.1); length of dorsal shield 690–860 μm	***Pachylaelaps pectinifer* (G. & R. Canestrini, 1882)**
20	Sperm induction system with short, conical to cylindrical, and evenly sclerotized structures	**21**
–	Sperm induction system not detectable or with normal, elongated and tubiform structures	**24**
21	Lateromarginal and ventral soft integument with 9–10 pairs of setae; cheliceral digits slim and elongated: subdistal and submedial tooth of movable digit small, subequal in size and with well separated position; length of dorsal shield 680–800 μm	***Pachylaelaps resinae* Karg, 1971**
–	Lateromarginal and ventral soft integument with 15–16 pairs of setae; cheliceral digits relatively shorter: movable digit with more adjacent subdistal and submedial tooth, submedial tooth distinctly larger than small subdistal tooth	**22**
22	Smaller species with dorsal shield under 850 μm in length (sternal shield less than 275 μm in length, genitiventral shield less than 305 μm in width); transversal curved sculptural line on sternal surface between setae st2 discontinuous medially; length of dorsal shield 750–800 μm	***Pachylaelaps terreus* Mašán, 2007**
–	Larger species with dorsal shield more than 850 μm in length (sternal shield more than 275 μm in length, genitiventral shield more than 305 μm in width); transversal curved sculptural line on sternal surface continuous	**23**
23	Sclerotized structures with sperm ductus stout, widened basally and completely abutting inner surface of coxae IV, and relatively short (with well separate apices); length of dorsal shield 880–1,022 μm	***Pachylaelaps insularis* Berlese, 1920**
–	Sclerotized structures with sperm ductus slim, narrow, and relatively long; length of dorsal shield 950–1,140 μm	***Pachylaelaps imitans* Berlese, 1920**
24	Smaller species with dorsal shield under 950 μm in length; tubiform structures of sperm induction system evenly sclerotized and relatively shorter (with their apices sufficiently separated); dorsolateral and ventral soft integument with 11 pairs of setae	***Pachylaelaps pyrenaicus* sp. n.**
–	Larger species with dorsal shield between 1,150 and 1,400 μm in length; tubiform structures not detectable (unsclerotized or absent), or unevenly sclerotized and obviously elongate, with adjacent apical or distal sections; apical or distal section of tubes more sclerotized than basal part; dorsolateral and ventral soft integument with at least 13 pairs of setae	**25**
25	Tubiform structures well developed, relatively long	**26**
–	Tubiform structures not detectable	**28**
26	Tubiform structures penis-like, straight or slightly curved, directed anteriorly, with slightly broadened base and more sclerotized tip; length of dorsal shield 1,185–1,330 μm	***Pachylaelaps armimagnus* Mašán, 2007**
–	Tubiform structures more elongated, worm-like to saccule-like, well broadened basally, strongly curved and directed posteriorly	**27**
27	Distal portions of tubiform structures relatively wide, saccule-like, closely adjacent, and uniformly sclerotized; lateromarginal and ventral soft integument with 13–14 pairs of setae; length of dorsal shield 1,320–1,350 μm	***Pachylaelaps sacculimagnus* Mašán, 2007**
–	Distal portions of tubiform structures narrow, worm-like, well distant, and with thickened terminal sclerotization; lateromarginal and ventral soft integument with 15 pairs of setae; length of dorsal shield 1,180–1,310 μm	***Pachylaelaps troglophilus* Willmann, 1940**
28	Lateromarginal and ventral soft integument with increased number of 20–21 pairs of setae; genitiventral shield relatively narrower (length/width 1.08–1.19); length of dorsal shield 1,245–1,300 μm	***Pachylaelaps ensifer* Oudemans, 1904**
–	Lateromarginal and ventral soft integument with 15 pairs of setae; genitiventral shield relatively wider (length/width 0.96–1.05); length of dorsal shield 1,190–1,400 μm	***Pachylaelaps carpathimagnus* Mašán, 2007**

### Key to European species of the genus *Pachylaelaps* (males)

**Table:** 

1	Dorsal setae J5 well developed, needle-like; posterolateral margins of dorsal shield with one pair of slit-like poroid structures, gdS4 (subgenus *Longipachylaelaps* Mašán, 2007)	**2**
–	Setae J5 vestigial; posterolateral margins of dorsal shield with two pairs of slit-like poroid structures, gdZ1 and gdS4 (subgenus *Pachylaelaps* s. str.)	**18**
2	Apex of cheliceral spermatodactyl with special horseshoe-like process; length of dorsal shield 810 μm	***Pachylaelaps virago* Berlese, 1920**
–	Apex of spermatodactyl regularly formed, never with additional process	**3**
3	Cheliceral spermatodactyl wider, with obvious basal or medial expansion and narrow distal section	**4**
–	Spermatodactyl narrower, sword-like to stiletto-like, with almost parallel lateral margins in medial section and moderately tapered distal section	**7**
4	Cheliceral spermatodactyl widened in basal section and relatively longer (spermatodactyl length/movable digit length 2.9–3.3); dorsal setae J5 less than two times longer than setae J4	**5**
–	Spermatodactyl widened in medial section and relatively shorter (spermatodactyl length/movable digit length 1.8–2.3); setae J5 at least two times shorter than setae J4	**6**
5	Dorsal setae J5 and J4 subequal in length; length of dorsal shield unknown	***Pachylaelaps pulsator* Hirschmann & Krauss, 1965**
–	Dorsal setae J5 about 1.5 times shorter than setae J4; length of dorsal shield unknown	***Pachylaelaps longicrinitus* Hirschmann & Krauss, 1965**
6	Dorsal setae J5 less shortened, about 2–3.5 times shorter than setae J4; two petal-like palptibial projections basally fused; length of dorsal shield 670–735 μm	***Pachylaelaps distinctus* Mašán, 2007**
–	Setae J5 more shortened, about 4–7 times shorter than setae J4; two petal-like palptibial projections free; length of dorsal shield 750 μm	***Pachylaelaps squamifer* Berlese, 1920**
7	Petal-like palptibial projections smaller, shorter than cross-sectional radius of palptibia	**8**
–	Petal-like palptibial projections larger, longer than cross-sectional radius of palptibia	**11**
8	Dorsal setae J4 and J5 subequal or only negligibly differing in length; dorsocentral setae shorter: setae J1 with tips reaching between insertions of setae J1 and J2	**9**
–	Dorsal setae J4 at least 1.5 times longer than setae J5; dorsocentral setae longer: setae J1 with tips reaching or overlapping the insertions of setae J2	**10**
9	Cheliceral spermatodactyl laterally flattened, sword-like, shorter (less than two times the movable digit); most dorsal setae short: z6 < z6‒Z1, s4 < s4‒s5, Z2 < Z2‒Z3; length of dorsal shield 470–510 μm	***Pachylaelaps brevipilis* sp. n.**
–	Spermatodactyl tubular, slightly sinuous, spear-shaped, longer (about three times the movable digit); most dorsal setae long: z6 > z6‒Z1, s4 > s4‒s5, Z2 > Z2‒Z3; length (mean) of dorsal shield 836 μm	***Pachylaelaps silviae* Moraza & Peña, 2005**
10	Cheliceral spermatodactyl widest in distal section; length of dorsal shield unknown	***Pachylaelaps granulifer* Hirschmann & Krauss, 1965**
–	Spermatodactyl widest in basal section; length of dorsal shield unknown	***Pachylaelaps gibbosus* Hirschmann & Krauss, 1965**
11	Two palptibial projections with parallel contiguous margins and adjacent apices	**12**
–	Two palptibial projections with divergent contiguous margins and apices well separated	**13**
12	Larger palptibial projection with widely rounded anterior margin; dorsal setae J5 38–51 μm long, about 1.5–2.5 times shorter than setae J4 (70–92 μm long); length of dorsal shield 645–735 μm	***Pachylaelaps longulus* Willmann, 1938**
–	Larger palptibial projection regularly tapered and with obtusely pointed apex; setae J5 about 30 μm long, about four times shorter than setae J4 (120–130 μm long); length of dorsal shield 710–780 μm	***Pachylaelaps sublongisetis* Koroleva, 1977**
13	One of the palptibial projections with needle-like process on distal margin	**14**
–	Palptibial projections never with needle-like process on distal margin	**15**
14	Distal margin of larger palptibial projection irregular, with two apices: anteriorly directed apex needle-like, laterally situated apex expanded and widely rounded; cheliceral spermatodactyl wider, with small subapical incision; length of dorsal shield 640–715 μm	***Pachylaelaps carpathicus* Mašán, 2007**
–	Distal margin of larger palptibial projection regularly curved, with one needle-like apex directed laterally; spermatodactyl narrower, with regularly tapered apex; length of dorsal shield 870–950 μm	***Pachylaelaps bocharovae* Koroleva, 1978**
15	Cheliceral spermatodactyl relatively longer (spermatodactyl length/movable digit length 1.8–2.4)	**16**
–	Spermatodactyl relatively shorter (spermatodactyl length/movable digit length 1.6–1.8)	**17**
16	Terminal hook of cheliceral fixed digit bifid; cheliceral spermatodactyl relatively shorter (spermatodactyl length/movable digit length 1.8–2); length of dorsal shield 745–900 μm	***Pachylaelaps perlucidus* Mašán, 2007**
–	Terminal hook of cheliceral fixed digit simple; spermatodactyl relatively longer (spermatodactyl length/movable digit length 2.2–2.4); length of dorsal shield unknown	***Pachylaelaps conifer* Hirschmann & Krauss, 1965**
17	Dorsal setae relatively longer: setae J3 with tips reaching to the bases of setae J5; cheliceral spermatodactyl relatively shorter (spermatodactyl length/movable digit length 1.6); length of dorsal shield unknown	***Pachylaelaps decipiens* Hirschmann & Krauss, 1965**
–	Dorsal setae relatively shorter: setae J3 with tips reaching between the bases of setae J3 and J5; spermatodactyl relatively longer (spermatodactyl length/movable digit length 1.8); length of dorsal shield unknown	***Pachylaelaps hestulifer* Hirschmann & Krauss, 1965**
18	Tarsus II with one spur-like distal seta (pl1)	**19**
–	Tarsus II with two spur-like distal setae (pl1, pl2)	**23**
19	Palptibial projections wider, each with widely rounded apex; cheliceral spermatodactyl with at least one small lobe-like convexity situated on ventral proximal margin	**20**
–	Palptibial projections narrower, at least one of them with needle-like apex; cheliceral spermatodactyl with straight margins, without lobe-like convexities on its margins	**21**
20	Two slit-like poroid structures on posterolateral dorsal surface well separated: gdZ1 situated between setae Z1–Z2 (close to Z2) and gdS4 between setae S4–S5 (close to S4); length of dorsal shield 760–840 μm	***Pachylaelaps denticulatus* Hirschmann & Krauss, 1965 *sensu* Koroleva, 1977**
–	Two slit-like poroid structures with more adjacent position on posterolateral dorsal surface: gdZ1 and gdS4 situated close to setae Z2	***Pachylaelaps pyrenaicus* sp. n.**
21	Cheliceral spermatodactyl with two small lobe-like convexities situated on dorsal distal margin and ventral proximal margin; length of dorsal shield 740 μm	***Pachylaelaps terreus* Mašán, 2007**
–	Spermatodactyl with one small lobe-like convexity situated on ventral proximal margin	22
22	Larger species, dorsal shield more than 750 μm in length; length of dorsal shield 980 μm	***Pachylaelaps imitans* Berlese, 1920**
–	Smaller species, dorsal shield less than 750 μm in length; length of dorsal shield 610–665 μm	***Pachylaelaps resinae* Karg, 1971**
23	Palptibia smooth, without projections	**24**
–	Palptibia with projections	**25**
24	Openings of slit-like poroids gdZ1 and gdS4 closely adjacent; projection on genu II small, subconical, with thin and rounded apex; cheliceral spermatodactyl about 1.5 times longer than movable digit; length of dorsal shield 700–830 μm	***Pachylaelaps littoralis* Halbert, 1915**
–	Openings of slit-like poroids gdZ1 and gdS42 relatively separate; projection on genu II robust, subcylindrical, with flat to truncate apex; spermatodactyl about two times longer than movable digit; length of dorsal shield 650–770 μm	***Pachylaelaps pectinifer* (G. & R. Canestrini, 1882)**
25	Smaller species with dorsal shield less than 1,000 μm in length; palptibia with two projections; cheliceral spermatodactyl relatively shorter (spermatodactyl length/movable digit length about 1.6); length of dorsal shield 825–840 μm	***Pachylaelaps insularis* Berlese, 1920**
–	Larger species with dorsal shield more than 1,000 μm in length; palptibia with 2–4 projections; cheliceral spermatodactyl relatively longer (spermatodactyl length/movable digit length more than 2.5)	**26**
26	Palptibia thickened: palptibial petal-like projections well developed and sclerotized, longer than cross-sectional radius of palptibia; proximal section of cheliceral spermatodactyl relatively wide and with punctate ornamentation on surface	**27**
–	Palptibia normal: palptibial petal-like projections weakly developed and sclerotized, shorter than cross-sectional radius of palptibia; proximal section of spermatodactyl relatively narrow and without punctation	**29**
27	Palptibia with two petal-like projections and a setiform structure, smaller petal-like projection with spinous apex; cheliceral spermatodactyl with small convexity on ventral proximal margin; length of dorsal shield 1,080–1,170 μm	***Pachylaelaps troglophilus* Willmann, 1940**
–	Palptibia with three petal-like projections (one of them with spinous apex) and a setiform structure; spermatodactyl without small convexity on ventral margin	**28**
28	Setiform structure associated with palptibial projections simple, tenuous and tubular; cheliceral spermatodactyl with regularly convergent lateral margins, lanceolate in subdistal part; length of dorsal shield 1,245–1,255 μm	***Pachylaelaps armimagnus* Mašán, 2007**
–	Setiform structure associated with palptibial projections flattened, plank-like, bifurcate apically, with two sharp points; spermatodactyl knife-like, with almost parallel lateral margins, slight subapical narrowing and rostrum-like tip; length of dorsal shield 1,230–1,360 μm	***Pachylaelaps sacculimagnus* Mašán, 2007**
29	Palptibia with two separate or fused scale-like projections, the projections with rounded or obtusely pointed distal margin; length of dorsal shield 1,235–1,245 μm	***Pachylaelaps ensifer* Oudemans, 1904**
–	Palptibia with four scale-like projections, the largest lateral projection hook-shaped subapically, sharply pointed; length of dorsal shield 1,170–1,325 μm	***Pachylaelaps carpathimagnus* Mašán, 2007**

## Supplementary Material

XML Treatment for Pachylaelaps

XML Treatment for Pachylaelaps (Pachylaelaps) pyrenaicus

XML Treatment for Pachylaelaps (Longipachylaelaps) brevipilis
